# Intra- and Extracellular Effector Vesicles From Human T And NK Cells: Same-Same, but Different?

**DOI:** 10.3389/fimmu.2021.804895

**Published:** 2021-12-23

**Authors:** Marcus Lettau, Ottmar Janssen

**Affiliations:** ^1^ Molecular Immunology, Institute of Immunology, University Hospital Schleswig-Holstein, Kiel, Germany; ^2^ Department of Internal Medicine II, Unit for Hematological Diagnostics, University Hospital Schleswig-Holstein, Kiel, Germany

**Keywords:** cytotoxic T cells, natural killer cells, lysosome-related effector organelles, exosomes, microvesicles, multivesicular bodies

## Abstract

Cytotoxic T lymphocytes (CTL) and Natural Killer (NK) cells utilize an overlapping effector arsenal for the elimination of target cells. It was initially proposed that all cytotoxic effector proteins are stored in lysosome-related effector vesicles (LREV) termed “secretory lysosomes” as a common storage compartment and are only released into the immunological synapse formed between the effector and target cell. The analysis of enriched LREV, however, revealed an uneven distribution of individual effectors in morphologically distinct vesicular entities. Two major populations of LREV were distinguished based on their protein content and signal requirements for degranulation. Light vesicles carrying FasL and 15 kDa granulysin are released in a PKC-dependent and Ca^2+^-independent manner, whereas dense granules containing perforin, granzymes and 9 kDa granulysin require Ca^2+^-signaling as a hallmark of classical degranulation. Notably, both types of LREV do not only contain the mentioned cytolytic effectors, but also store and transport diverse other immunomodulatory proteins including MHC class I and II, costimulatory and adhesion molecules, enzymes (i.e. CD26/DPP4) or cytokines. Interestingly, the recent analyses of CTL- or NK cell-derived extracellular vesicles (EV) revealed the presence of a related mixture of proteins in microvesicles or exosomes that in fact resemble fingerprints of the cells of origin. This overlapping protein profile indicates a direct relation of intra- and extracellular vesicles. Since EV potentially also interact with cells at distant sites (apart from the IS), they might act as additional effector vesicles or intercellular communicators in a more systemic fashion.

## Introduction – Secretory Vesicles in Hematopoietic Cells

Several cell types of the hematopoietic lineage utilize lysosome-related effector vesicles (LREV) with an inducible secretory potential as highly specialized vehicles for the storage and mobilization of cell-type-specific regulator or effector molecules. Examples include antigen-presenting cells (APC) such as macrophages, dendritic cells or B cells containing vesicular MHC compartments for the storage, processing and presentation of antigen-loaded MHC-molecules (1), basophils and mast cells secreting histamine and serotonin in response to Fc receptor ligation or chemotactic agents (2), and platelets releasing effector molecules such as serotonin and P-selectin for blood coagulation (3). In cytotoxic T cells (CTL) and Natural Killer (NK) cells, so-called lytic granules comprise lysosomal storage and effector compartments that – among others - carry pore-forming perforin (PRF) or granulysin (GNLY), proteolytically active granzymes (GRZs), and apoptosis-inducing death ligands such as FasL to warrant an effective target cell lysis (4–7). Thereby, CTL and NK cells form the prominent effector cell populations of the immune system to control virus-infected or tumor cells. Although they utilize a similar arsenal of effector proteins, the mode of induction of granule maturation and release differs substantially (6). CD8^+^ CTL are activated *via* their antigen-specific T-cell receptors (TCR) by antigen-presenting cells (APC), which present viral or tumor-associated antigens on MHC class I molecules. Upon initial antigen contact, CTL differentiate to more potent cytotoxic effectors by consecutively replenishing effector molecules to their lytic granules. In contrast, γδ T cells are very efficient CTL containing comparable levels of effectors as CD8^+^ CTL without being MHC-restricted. Therefore γδ T cells may serve as cellular constituents for novel approaches in cancer immunotherapy (8). NK cells lack antigen-specific receptors, but sense reduced MHC expression or stress-induced ligands on transformed cells by a collection of inhibitory and activating receptors. For NK cells, it is believed that the lytic granules become already fully equipped during differentiation and maturation (9–11).

Notably, the initial steps of activation and granule maturation do not comprise the main focus of this article. However, one should keep in mind that initiation of differentiation, maturation and release do not rely on ligation of single receptors resulting in a uniform intracellular signaling cascade, but – especially regarding the individual cell populations - are far more complex than described above. To mention just a few parameters that may alter a respective trigger after cell-cell-contact: ligation of costimulatory and accessory molecules, cytokine- and chemokine-receptors, the formation of membrane platforms (e.g. lipid raft or tetraspanin platforms), tonic signals and the abundance of signaling enzymes or adapter proteins define the individualized outcome on a single cell basis [reviewed in (12, 13)].

Although the timing and modes of activation and maturation are different, CTL and NK cells nonetheless use a similar equipment of cytotoxic effector proteins which are stored in LREV that have been termed “secretory lysosomes”, indicating that these bi-functional organelles combine lysosome-associated degrading functions with storage and secretory properties (5, 14). At least a portion of these organelles carries typical “lysosome associated membrane proteins” (LAMPs), displays a low pH and contains hydrolases that are also characteristic for conventional lysosomes (14, 15). In one subtype of these vesicles, referred to as dense granules, the low pH ensures a tight packaging and transient inactivation of effector molecules such as PRF or GRZs which is regulated for example by serglycins (16). Besides LAMPs, the organelle membranes of LREV from activated T cells (or NK cells) carry other transmembrane molecules with crucial immune functions, including the death factor FasL (CD178) (17), the negative co-stimulator CTLA-4 (CD152) (18), or dipeptidylpeptidase 4 (CD26/DPP4) (19). This might indicate that discrete components of secretory lysosomes are produced or mobilized in a context-dependent manner for specific effector functions, down-modulation of immune responses or differentiation.

Previously, we reviewed the knowledge and ideas about different lysosome-associated membrane, effector or cargo proteins, also in the context of diseases associated with loss of function mutations in lysosomal transport regulators (6, 15). Here, we focus on more recent developments and novel aspects regarding the mechanistic peculiarities of individual populations of LREV in T and NK cells. We address the distribution of vesicles in different T-cell subsets, their distinct signal requirements for degranulation and a potential relation of intracellular LREV and extracellular vesicles. Notably, besides classical cytotoxic cells (i.e. CD8^+^ αβ T cells, γδ T cells, NK cells), also CD4^+^ T cells develop secretory granules when activated and expanded *in vivo* or *in vitro* (20, 21). However, as we have pointed out in several studies, classical cytotoxic cells carry most of the mentioned vesicle-associated proteins in higher abundance than cytotoxic CD4^+^ T cells (7, 19, 22).

## Enrichment and Analysis of Lysosome-Enriched Effector Vesicles

Strategies for the enrichment of intact intracellular organelles rely on a mild cell disruption by controlled dounce or carbid balch homogenization, followed by various steps of differential and density gradient centrifugations [see refs (23) and (6) for more information]. We routinely employ an adapted iodixanol-based protocol to enrich intact organelles from T lymphocytes and NK cells and analyzed putative marker proteins for intracellular compartments by Western blotting. Notably, as substantiated by electron microscopy, LREV stably segregated into light and dense vesicles which could be clearly distinguished from mitochondria.

We started our LREV profiling with enriched organelles from *in vitro* expanded primary human NK cells and leukemic NK-cell lines (24). Based on the detection of FasL as a marker for secretory lysosomes [as suggested by Griffiths and co-workers (25)], in light vesicles of primary NK cells (corresponding to fraction 2 of the discontinuous density gradient), we focused on the proteome profiling of this vesicular entity and provided a first comprehensive proteome map of ‘secretory lysosomes’ from NK cells (24). We found that the overall protein repertoire within this organelle fraction from different cellular sources was rather similar and biological replicates of individual preparations yielded nearly identical results. Nonetheless, we noted striking differences in the individual abundance of functionally relevant proteins including MHC molecules, cathepsins, cytokines such as IL-16, and GRZA and GRZB in preparations from the individual cell populations, indicating a clonotypic distribution of lysosomal proteins in primary and leukemic NK cells (24). In addition, this study also showed that proteome analyses based on individual cell lines cannot be simply extrapolated to define the lysosomal proteome of their non-transformed counterparts.

Regarding the distribution of GRZB, we were puzzled by the fact that in isolates from primary NK cells, the major portion of GRZB was unexpectedly not associated with FasL-containing light vesicles in fraction 2, but rather appeared in heavier fractions and especially in dense granules of fraction 6. In contrast, GRZB was present in high amounts in almost all fractions isolated from YTS cells and in the lighter fractions (1–4) from NKL cells (with the highest abundance again in fraction 2). Notably, the differential distribution of FasL and GRZB in enriched vesicles from primary NK cells provided first biochemical evidence for the presence of distinct or at least separable effector organelles in NK cells.

## Evidence for Two Species of LREV in Human T and NK Cells

We also investigated the proteome of enriched organelles from PHA-activated human T-cell blasts (26, 27). Individual fractions were analyzed by Western blotting, e.g. for Cadherin as a plasma membrane marker, Bip/Grp78 as an ER marker, and CoxIV as a marker for mitochondria. CD63, LAMP-1 (CD107a), cathepsin D and Vti-1b were used to detect lysosomal compartments, and - more specifically - FasL, PRF, GRZB and GNLY served as markers for secretory lysosomes (26). Since FasL and CD63 were enriched in light fraction 2 vesicles, which also contained other lysosomal proteins such as LAMP-1 or cathepsin D, these putative secretory lysosomes were subjected to MS-based proteome profiling (26). The classification of the approximately 400 identified proteins revealed that 70% had been assigned to lysosome-related organelle compartments in previous studies (26). During these analyses, however, it became evident that also in T-cell blasts, GRZB was more or less exclusively found in dense granules separated in fraction 6 of the gradients. Interestingly, we noticed that the 15 kDa GNLY precursor was detected in fraction 2 vesicles whereas the mature 9 kDa GNLY was prominent in dense granules of fraction 6 (27). Nonetheless, also dense granules in fraction 6 proved to be lysosome-related organelles since two third of the identified proteins had been annotated as “lysosome-associated”. Therefore, the fraction of dense granules reflected a stable second lysosomal effector compartment with an enrichment of PRF, GRZB and mature 9 kDa GNLY (27).

Importantly, preparations of individual LREV from PHA-activated T-cell blasts of different donors were extremely homogeneous in their overall protein content and regarding the distribution or segregation of proteins to light or dense vesicles. By directly comparing fraction 2 and fraction 6 vesicles, we were able to verify the selective distribution not only for the key effector proteins FasL, GRZB, PRF and GNLY, but also for other cargo proteins (27). Moreover, the differential association of the two granule types with adapter proteins such as Nck or WASp, cytoskeletal proteins including actin, actinin or myosin and the GTPase dynamin indicated that the two compartments might be linked to or mobilized by distinct cytoskeletal elements (i.e. β-actin for fraction 2 and myosin IIa for fraction 6) (6, 27). These observations lead to the hypothesis that mobilization of distinct LREV might follow different activation- and cytoskeleton-dependent transport routes.

## Differential Exocytosis of T-Cell-Derived LREV

Degranulation or exocytosis of secretory vesicles is regarded to be essential for the formation of the cytotoxic immunological synapse (IS) (17). In T cells, ligation of the TCR usually triggers a Ca^2+^ influx and polarization of the “microtubule organizing center” (MTOC) towards the IS. Tubulin-associated transport is mediated by dynein and might also involve actin-dependent movement *via* myosin IIa in the F-actin rich cell periphery. Subsequent fusion with the plasma membrane relies on different addressing factors including “soluble N-ethylmaleimide-sensitive factor attachment receptors” (SNAREs) (28). Membrane fusion and degranulation result in the release of effector proteins (e.g. PRF and GRZs) into the intracellular synaptic space and the local appearance of secretory lysosome-associated membrane proteins (i.e. FasL) on the cell surface (29, 30). Importantly, according to the model proposed by Griffiths and co-workers, the FasL molecule had been regarded as a characteristic transmembrane marker protein of secretory lysosomes in T and NK cells, and supposedly was associated with the same SL-compartment as PRF and GRZs (5, 14, 31). This all-in-one model, however, was challenged by our observations and by studies of He, Ostergaard and colleagues who reported that degranulation and release of GRZs and PRF would require Ca^2+^-dependent signals whereas FasL surface appearance was seen in a Ca^2+^-independent manner (32, 33). Moreover, Kassahn and colleagues revealed that FasL- or PRF/GRZ-release require distinct signal thresholds and cytoskeletal elements (34).

It was thus proposed that cytotoxic effector proteins might be either stored in physically distinct LREV entities or recruited *via* different routes from a common storage compartment. Considering that secretory lysosomal compartments mature in or from endosome-derived multivesicular bodies (MVB), one could imagine that a portion of the MVB might fuse with late endosomes to give rise to either lysosome-derived dense granules (primarily containing GRZs or PRF) or to secretory lysosomes that contain FasL (30). This would be compatible with earlier microscopic analyses showing at least a partial co-localization of FasL, PRF and GRZB and other lysosomal markers, and at the same time account for the more recent observations of discrete lysosomal effector organelles. Nonetheless, until today it remains to be elucidated how exactly the mobilization and release of PRF- and GRZ-loaded granules is mechanistically segregated from the surface appearance of FasL.

In order to shed light on the mobilization and release of individual effector proteins from cytotoxic T cells, we utilized chemical activators or inhibitors to circumvent the complex signaling following TCR-ligation. Employing phorbol ester and calcium ionophore alone or in combination, we were able to demonstrate that signal requirements for the mobilization of individual effectors differ substantially (22). We had shown earlier that in response to PKC activation and calcium mobilization, FasL displays a biphasic surface appearance with a first maximum after ten minutes representing the mobilization of pre-stored molecules and a second maximum after 90-120 minutes based on *de novo* synthesis (35). Notably, also upon stimulation with phorbol ester alone, FasL was rapidly mobilized to the cell surface resulting in the first peak whereas no second increment was observed (22). When the same cells were treated with ionomycin alone, two phases of FasL expression were detected, but never reached the amplitude as in the presence of additional phorbol ester. Since especially the first maximum was much less pronounced upon ionomycin only stimulation, we concluded that – as suggested before by Kassahn and colleagues (34) - preformed FasL can be mobilized in a PKC-dependent and calcium-independent manner (22). Of note, similar kinetics were observed in all tested cytotoxic T-cell populations (i.e. cytotoxic CD8^+^ T cells or γδ T cells and *in vitro* expanded CD4^+^ T cells).

**Figure 1:**
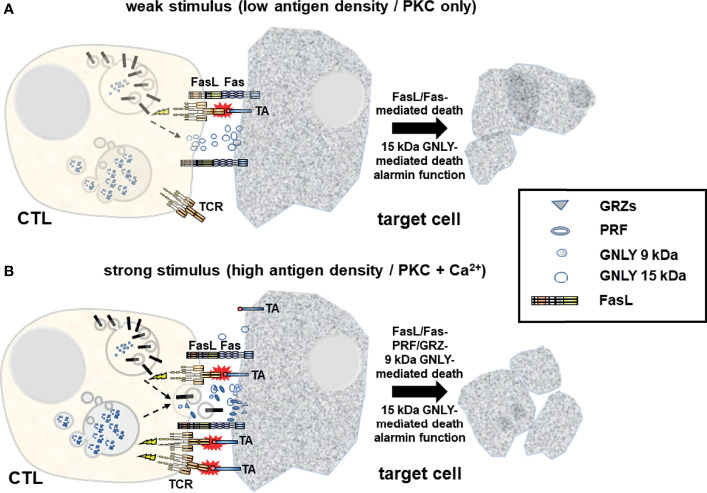
Upon target cell encounter LREV/MVB are mobilized to the site of intercellular contact where they fuse with the membrane to release cytotoxic effector molecules into the forming immunological synapse and to expose transmembrane death factors (e.g. FasL) locally on the cell surface. **(A)** At weaker stimuli (e.g. low antigen density on the target cell) and in the absence of Ca^2+^ signaling, FasL and 15 kDa GNLY are mobilized from light LREV in a PKC-dependent non-classical degranulation process exposing FasL locally on the plasma membrane and releasing 15 kDa GNLY into the immunological synapse. FasL induces cell death in Fas^+^ target cells. At the same time, 15 kDa GNLY might act as a supporter of cytolysis but also as an alarmin to recruit more immune cells to the site for immunosurveillance. It is unclear to date whether FasL^+^ exosomes are also released in response to weak stimuli. **(B)** In response to strong stimuli (e.g. high tumor antigen (TA) density), additional Ca^2+^ signaling triggers the release of prestored soluble cytotoxic effector molecules including PRF, GRZs and 9 kDa GNLY from dense MVBs. This process is generally referred to as classical degranulation. At the same time, the death factor FasL and the 15 kDa GNLY are also mobilized. The composition of cytotoxic effectors released in a given situation might thus dictate the characteristics of the cell death program executed in different target cells with respect to immunogenicity and further surveillance or clearance.

Further analyses using phorbol ester and calcium ionophore and a variety of inhibitors addressing different steps of cytoskeleton dynamics revealed that non-transformed human T cells do not only possess at least two distinct species of LREV, but that these individual sets of LREV are indeed differentially regulated with respect to signaling requirements and mechanisms for their transport and mobilization (22). This suggested that a given T cell might in fact sense which arsenal of effector vesicles or proteins it needs to mobilize to kill a given target cell with highest specificity and efficacy while minimizing collateral damage (**Figure 1**).

This hypothesis is in line with an earlier study by Shankar and co-workers (36) who investigated the biological relevance of PRF- and FasL-mediated cytolytic pathways of CD8^+^ CTL for cancer immunotherapy in a murine renal cell carcinoma model with tumor cells expressing influenza viral hemagglutinin (Renca-HA) as a defined surrogate antigen. They observed that Renca-HA injection killed FasL-defective gld/gld mice but not PRF−/− and Fas-mutated lpr/lpr mice. However, blocking FasL *in vivo* inhibited tumor rejection in the latter mice. They also showed that established Renca-HA tumors were cleared more efficiently by adoptively transferred HA518–526-specific TCR-transgenic CTL utilizing FasL rather than PRF. Most importantly, mouse tumor cells expressing low levels of immunogenic peptide were preferentially lysed by FasL-mediated killing, whereas at higher peptide concentrations, the preference in effector pathway usage was lost and killing was achieved by cooperate action of FasL and PRF (36), indicating that at low concentrations of antigen, only FasL is mobilized whereas at high antigen-density, all granules are released.

Of note, the selection of cytotoxic effector molecules that ultimately exert cell death might dictate the characteristics of the subsequent immune response. Depending on the death-inducing stimulus and target cell-intrinsic factors, cell death can be either non-immunogenic or immunogenic. Immunogenic cell death is accompanied by the release of damage-associated molecular patterns (DAMPs) from dying cells, that in tumor malignancies results in the activation of tumor-specific immune responses and long-term antitumor immunity and thus plays a major role in immunosurveillance and immunotherapy. The type of cancer cell death also influences the local tumor environment by modulating levels of released inflammatory versus tolerogenic substances [reviewed in (37)]. As an example, a recent work employing human NK cells showed that the magnitude of calcium signaling and the relative concentrations of released PRF and GRZB dictated the apoptosis/necrosis ratio in target cells in *in vitro* assays (38).

## GNLY Variants as Markers for LREV Subsets and Differential Degranulation

Granulysin (GNLY) is a cationic cytotoxic effector protein of the saposin-like protein family present in CTL and NK cells (39). GNLY was found in cytolytic granules with PRF and GRZs and released *via* receptor-mediated degranulation (40). GNLY, however, comes in two flavors with distinct biological properties. The 15 kDa (full length) variant is regarded as a precursor for the short 9 kDa form which is generated by proteolytic processing at both termini from the long form (41). It was initially proposed that the 15 kDa GNLY lacks major cytotoxicity against bacterial and mammalian cells, but causes differentiation of monocytes to dendritic cells (42).

According to our observations, GNLY may in fact serve as a prototypic marker for the detection and analysis of different LREV species (7). GNLY is differentially distributed in individual subsets of T cells and NK cells. In T cells, GNLY expression goes in line with activation and maturation of LREV. In innate lymphocytes, such as NK cells and γδ T cells, constitutive GNLY expression might be more pronounced than in CD8^+^ CTL (7). This is in agreement with the in-depth mass cytometry profiling of human T and NK cells for patterns of differentiation and abundance of cytotoxic effectors (43) and also with the GNLY content analyses in decidual lymphocytes by Dominovic and colleagues (44) who reported that fresh decidual lymphocytes display inferior cytotoxic activity, and that after cell activation, the cytotoxic potential increases due to an accumulation of 9 kDa GNLY in PRF- and CD107a-containing vesicles.

In our studies on the differential liberation of LREV containing individual GNLY species (7), low level signaling e.g. induced by PKC activation through phorbol ester stimulation resulted in the selective mobilization of vesicles containing 15 kDa GNLY (and FasL). In contrast, strong signaling, e.g. triggered by phorbol ester and calcium ionophore, resulted in PKC activation and Ca^2+^ influx and the release of the full arsenal of effectors including light and dense granules with both GNLY species and associated FasL and GRZs and PRF. Along this line, a time-dependent processing and segregation of precursor and mature GNLY to different subcellular compartments had already been reported when GNLY was introduced as a cytotoxic effector molecule in 1997 (41). Both GNLY species were later found to be located in cytoplasmic granules, but only the 9 kDa mature GNLY was present in dense cytolytic granules. Moreover, it was suggested that the 15 kDa GNLY, which might lack cytotoxic activity and exert more immunomodulatory functions instead (42), is spontaneously secreted by CTL *via* a “non-exocytotic” (Ca^2+^-independent) pathway, whereas the 9 kDa cytolytic GNLY is released through Ca^2+^-dependent degranulation during target cell killing (39, 45). In terms of functional differences of the GNLY species, it was shown that the 15 kDa GNLY acts as an ‘alarmin’ in the inflamed extracellular space, and promotes the recruitment of macrophages (46) whereas the 9 kDa variant exerts major antimicrobial and cytotoxic effects when it is released together with PRF and GRZB (47). Accordingly, a selective and context-dependent induction and release of the different LREV including their functionally distinct cargo proteins might push immune responses into inflammatory or effector branches.

## Classical Ca^2+^-Dependent Degranulation Releases Proteolytically Active CD26/DPP4

CD26/DPP4 is a 110 kDa type 2 transmembrane glycoprotein that belongs to the S9 protease family of prolyl oligopeptidases (dipeptidylpeptidase 4, EC 3.4.14.5) (48). As a T-cell surface molecule, CD26/DPP4 has been implicated in the modulation of T-cell activation and proliferation (49). CD26/DPP4 on T cells interacts with caveolin-1 on antigen-presenting cells and thereby induces an increase in CD86 expression to facilitate T-cell co-stimulation (50). In turn, caveolin-1-mediated CD26/DPP4-ligation induces T-cell proliferation and NF-κB activation in a TCR/CD3-dependent manner (51). Moreover, CD26/DPP4-mediated co-stimulation of anti-CD3-activated CD8^+^ T cells enhances the cytotoxic effector function as compared to CD28-stimulation (52).

During our proteome analyses, we had noted that CD26/DPP4 is stored in different secretory granules of *in vitro* expanded T-cell blasts (26, 27). More recently, we could demonstrate that upon stimulation or target cell encounter, CD26/DPP4 is rapidly translocated to the cell surface of all cytotoxic lymphocyte populations in a Ca^2+^-dependent manner followed or accompanied by the release of proteolytically active soluble sCD26/DPP4 (19). Although the manifold effects of active CD26/DPP4 are far from being elucidated, we provided prime evidence that activated cytotoxic lymphocytes serve as a major source of sCD26/DPP4 and that classical degranulation governs sCD26/DPP4 release.

In pre-activated CD4^+^ and CD8^+^ αβ T cells, γδ T cells and NK cells, we found intracellular CD26/DPP4 preferentially, but not exclusively, in granules with GRZA, GRZB, PRF and GNLY (19). While the highest relative abundance of LREV-associated CD26/DPP4 was detected in NK cells, the varying co-localization measured by imaging flow cytometry might indicate a segregation to different compartments in individual T-cell subsets. Prior to our studies, CD26/DPP4 was mostly described as a plasma membrane-associated molecule while the subcellular localization had not been analyzed in depth. However, Fukui and colleagues had detected CD26/DPP4 in lysosomes of hepatocytes, endothelial cells and Kupffer cells in ultrathin sections of rat liver by electron microscopy before (53). Also, in pancreatic islets of pigs, immunoelectron microscopy revealed the presence of CD26/DPP4 in the secretory granules of A-cells (54). In 2007, Casey and colleagues identified CD26/DPP4 in a proteomic screen of enriched secretory lysosomes/cytotoxic granules from the NK lymphoma cell line YTS (55). Notably, based on an early finding that CD26/DPP4^+^ and CD26/DPP4^-^ T cells exhibit comparable levels of CD26/DPP4 mRNA and overall protein, Mattern and co-workers had already suggested an intracellular pool of CD26/DPP4 in T cells in 1995 (56).

Our recent analyses revealed that upon TCR ligation, pre-stored CD26/DPP4 is rapidly mobilized from intracellular storage granules in a strictly Ca^2+^-dependent fashion and thus follows the hallmarks of classical degranulation (19). In contrast to FasL expression, the activation-induced surface appearance of CD26/DPP4 was rather moderate. Instead, we detected proteolytically active sCD26/DPP4 in respective culture supernatants. Our studies therefore strongly supported earlier findings which suggested that lymphocytes might be contributing to the fairly high levels of sCD26/DPP4 which are detected in diverse body fluids such as serum, saliva, cerebrospinal and seminal fluid and bile. Notably, serum or plasma levels of sCD26/DPP4 protein and enzymatic activity are meanwhile regarded as indicators for immunodeficiency and/or increased lymphocyte activity in inflammation or cancer, respectively (57). Thus, it was recently demonstrated by Casrouge and co-workers that individuals with congenital lymphocyte-immunodeficiency displayed decreased sCD26/DPP4 serum levels that were normalized upon restoration of hematopoiesis (58). Moreover, sCD26/DPP4 serum levels in healthy controls or treated patients correlated with numbers of circulating lymphocytes. *In vitro* analyses revealed that T-cell stimulation increased the sCD26/DPP4 release. In a mouse model, infection with influenza virus resulted in elevated sCD26 serum levels that correlated with an increased frequency of antigen-specific CD8^+^ T cells (58). Of note, not only classical cytotoxic lymphocytes might release sCD26/DPP4. As an example, plasma DPP4 activity in Diabetes mellitus type 2 patients was attributed to the enhanced release of sCD26/DPP4 from circulating TH17 cells (59). Here, patient’s TH17 cells showed reduced surface expression of CD26/DPP4 that positively correlated with increased plasma DPP4 activity arguing for TH17 cells as a source for the elevated plasma sCD26/DPP4 abundance associated with Diabetes mellitus type 2.

It was proposed that sCD26/DPP4 originates from the proteolytic cleavage of full length CD26/DPP4 (59, 60) liberating the proteolytically active soluble form that lacks the intracellular region and the transmembrane domain. However, the protease(s) mediating sCD26/DPP4 release from cytotoxic cells remain(s) to be identified although the metalloproteases MMP1, MMP2 and MMP14 have been implicated in the liberation of sCD26/DPP4 from smooth muscle cells, MMP14 in the shedding from adipocytes (60) and the serine protease kallikrein 5 (KLK5) in the release from TH17 cells (59). In addition, besides shedding of transmembrane CD26/DPP4 from the cell surface, the enzyme might also be processed within intracellular storage granules to allow for the rapid release of sCD26/DPP4 upon mobilization and fusion of cytotoxic granules with the plasma membrane. Thus, Poulsen and colleagues suggested that CD26/DPP4 is stored as a soluble protein in secretory granules of pancreatic islet A-cells, because electron microscopy did not reveal a specific association with granule membranes (54). Although we did not detect truncated CD26/DPP4 in cellular lysates, an intracellular processing might well be induced by T-cell activation and precede degranulation.

However, additional studies are required to assess the cellular site, the molecular mechanism and the biological consequences of CD26/DPP4 processing and function in health and disease. Along this line, we have drafted a comprehensive review that summarizes the current views on CD26/DPP4 as a marker and modulator in non-transformed and malignant T cells (61).

## Conceptually, Intra- and Extracellular Vesicles Should Not Be Separated

It is meanwhile well appreciated that most if not all nucleated cells, including CTL and NK cells, secrete lipid enclosed extracellular vesicles (EVs) of different sizes and subcellular origin. According to the recently updated guidelines of the International Society for Extracellular Vesicles (ISEV, https://www.isev.org/), “extracellular vesicle” serves as a generic term for particles that are delimited by a lipid bilayer, that are naturally released from a cell and cannot replicate due to the absence of a functional nucleus (62). According to these guidelines, it is meanwhile commonly propagated that the smallest EVs, also termed exosomes, display sizes between approximately 30-150 nm in diameter and originate from multivesicular bodies (MVBs) of resting or activated cells. Microvesicles or microparticles have also been termed ectosomes and might be similarly small (starting at around 100 nm), but may also reach larger sizes up to 1 µm in diameter. In contrast to exosomes, these vesicles are released by plasma membrane protrusion and budding from activated or transformed cells and are thus regarded as a fingerprint of the donor cell with respect to surface decoration and luminal content. The third category of EVs are apoptotic bodies which are released from dying (apoptotic) cells. They can be of different size in the range of 50-5000 nm depending on the morphological changes and blebbing during programmed cell death (62). In an attempt to normalize the often somewhat unprecise EV nomenclature, ISEV has proposed the term small EVs to refer to vesicles of less than 200 nm in diameter, and large EVs for vesicles of more than 200 nm (63), although this does not entirely reflect the crucial differences in biogenesis.

Importantly, EVs might interact with cells in close proximity, but due to their stability in body fluids, also at more distant sites, where they might trigger specific receptor signaling in recipient cells, including ligation of cytokine-, co-stimulatory or death receptors. In addition, EVs might be also taken up by recipient cells by different means including membrane fusion, pinocytosis, phagocytosis, or clathrin-, caveolin- or lipid raft-mediated endocytosis (64, 65). The production of T cell-derived exosomes might be constitutive, but also inducible e.g. by TCR activation (66). Since the putative role of exosomes and their cargo proteins in the regulation of T cell-mediated immune responses and autoimmune diseases has been recently reviewed by Anel and co-workers (67) and Del Vecchio and colleagues comprehensively reviewed the role of extracellular vesicles in the interactions between NK cells and CD8^+^ CTL and tumor cells (68), we will focus on the potential relationship between intra- and extracellular vesicles in the following. The basis for the close relationship of intra- and extracellular vesicles is the common origin of secretory lysosomes and exosomes from cytoplasmic MVBs. In terms of biogenesis, both entities have been associated with molecules that govern MVB biogenesis including TSG101, ALIX or syntenin 1 (69). In fact, the recent comprehensive proteome analysis performed by Kugeratski and co-workers revealed that within EV populations, solely exosomes contain such MVB biogenesis markers, with syntenin 1 being highly abundant in exosomes but not in microvesicles or apoptotic bodies from different cellular sources (69).

## Common Biogenesis of LREV and Exosomes

**Figure 2:**
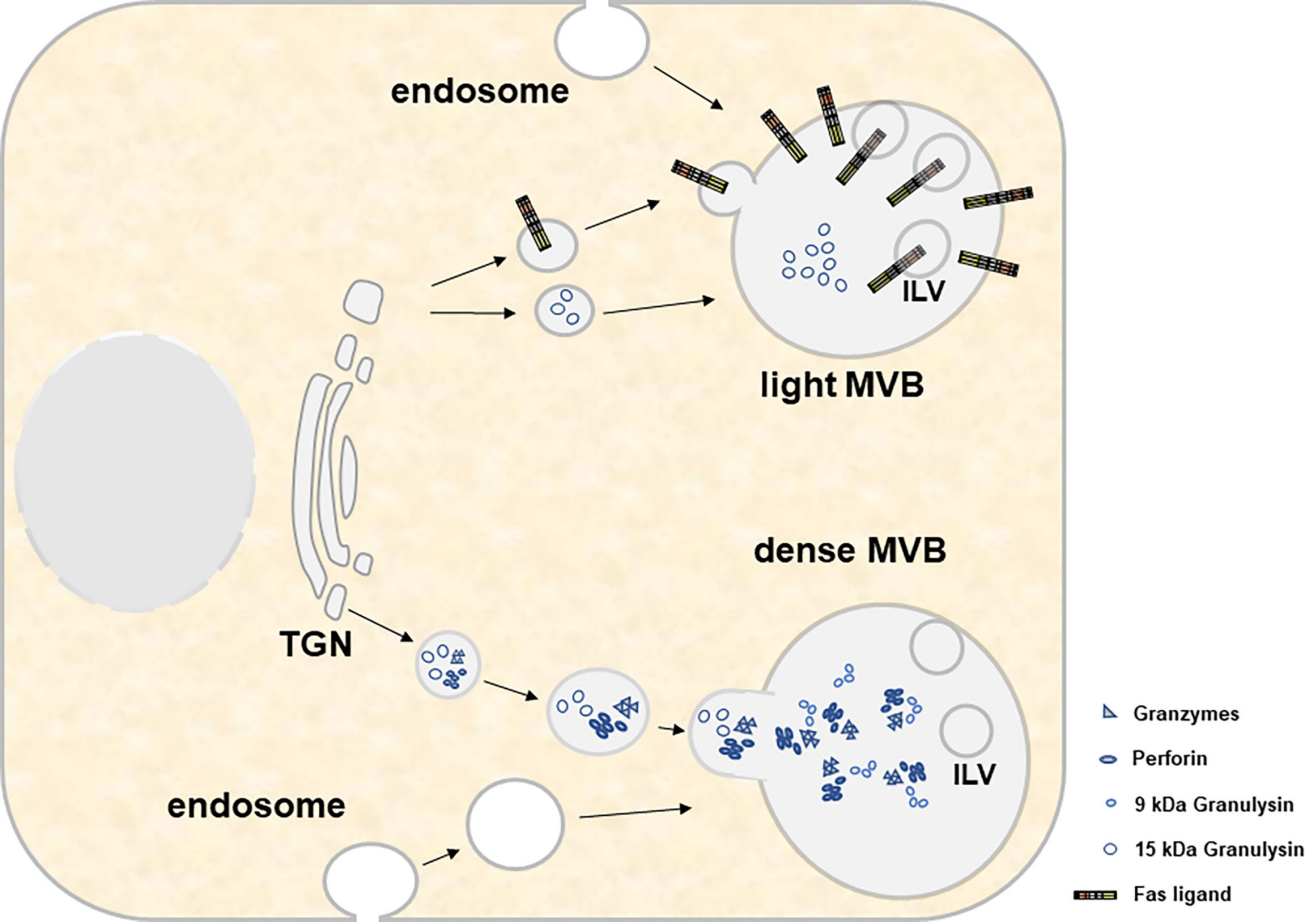
For both light and heavy MVBs, cytotoxic effector molecules and other constituent soluble proteins are transported from the trans-Golgi network. This transport involves several compartments, fusion events and organelle maturation steps that are not displayed in the present cartoon. Material transfer to MVBs involves transitory transport vesicles, early and late endosomes, recycling endosomes and/or endolysosomes/lysosomes. Moreover, it includes several maturation events such as the decrease of the pH, the formation of intraluminal vesicles (ILV) and the accumulation of cytolytic effector proteins within electron-dense cores. While light MVB are characterized by the presence of 15 kDa GNLY and FasL, dense MVB contain GRZs, PRF and 9 kDa GNLY.

If one compares the recent literature on the biogenesis of lysosome-related organelles (LRO) or secretory granules (70–73) and exosomes (74–78), respectively, common pathways, especially at the level of MVB formation and transport or release become more than evident. LRO include the lytic granules present in CTL and NK cells. These are likely modified lysosomes and occasionally contain a ring of intracellular vesicles surrounding a dense core that might directly derive from the trans-Golgi Network (TGN) and later fuse with multivesicular endosomes (MVEs) to form a dual functional hybrid organelle (70, 79). Apparently, different components of the biogenesis machinery are involved in the selective cargo loading of individual LRO subtypes [reviewed in (70)]. Moreover, in a complex scenario of endocytosis, recycling, fusion and delivery processes, lysosomes form as terminal compartments of the endocytic and autophagic pathways before they receive additional cargo from the TGN and/or *via* early or recycling endosomes that invaginate or recycle from the plasma membrane and form late endosomes or MVB (71). It is believed that such late endosomes/MVB fuse with lysosomes to form endolysosomes which might further mature to secretory granules or re-form back to lysosomes (71). Thus, the loading of secretory lysosomes might be achieved at various steps during the maturation process and ultimately determines the fate and function of the organelle (**Figure 2**).

**Figure 3:**
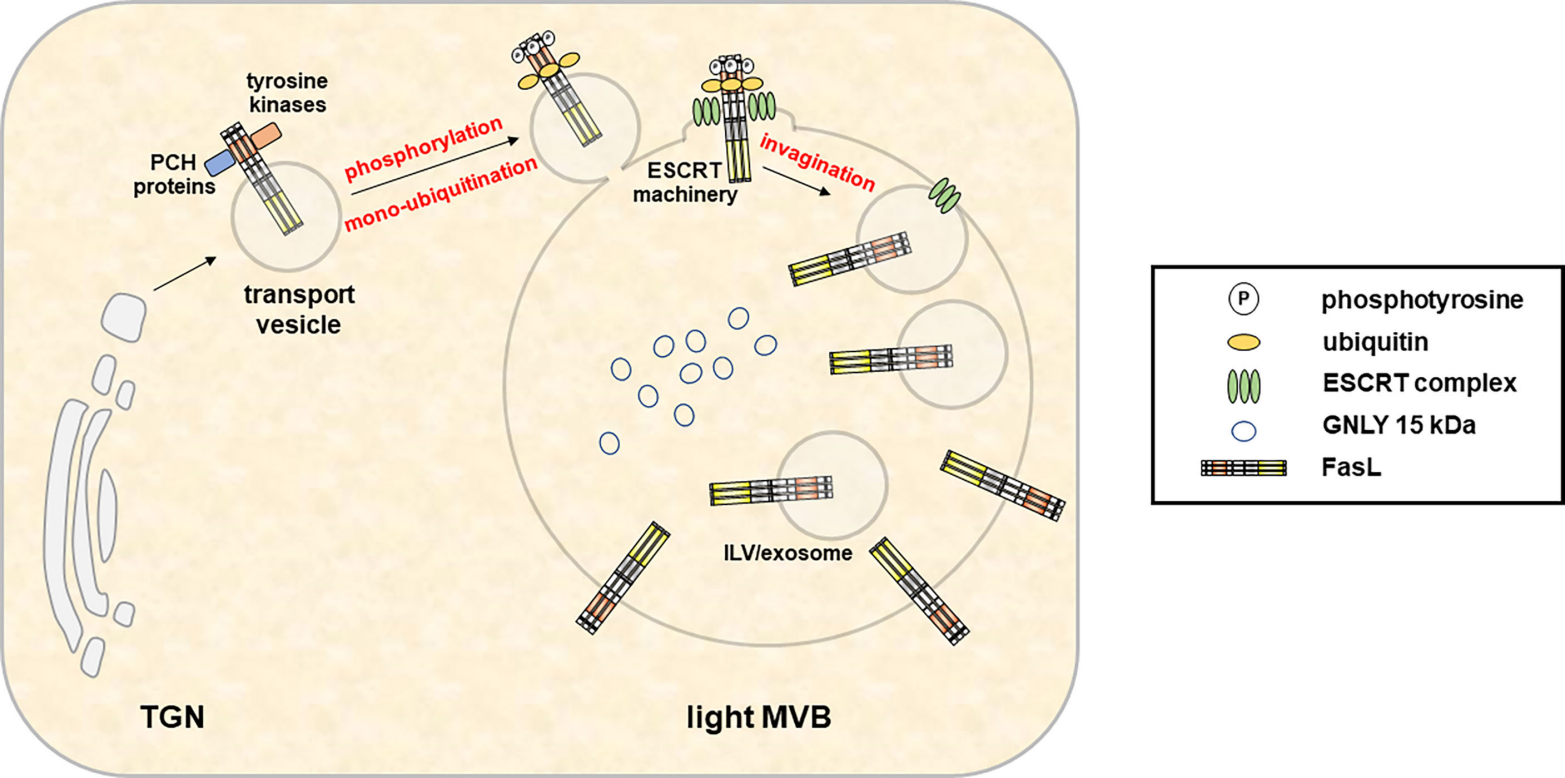
In hematopoietic cells, the type 2 transmembrane death effector FasL is sorted to intracellular MVB for storage and regulated release in response to appropriate stimuli. Fusion of the FasL-MVB with the plasma membrane results in the local exposure of FasL molecules present in the MVB outer membrane on the cell surface. Moreover, FasL localized in the membrane of ILV is released in association with exosomes. An extended proline-rich domain within the FasL cytoplasmic domain enables interactions with PCH proteins and Src kinases and is required for the sorting to MVBs. In addition, FasL localization also depends on phosphorylation of intracellular tyrosines. However, the exact sorting mechanisms and subcellular localizations of protein interactions and phosphorylation events are not defined yet. Subsequently, mono-ubiquitination of the FasL at intracellular lysines enables its sorting to ILV by the ESCRT machinery.

The loading and secretion of secretory lysosomes from CTL and NK cells with prominent effector molecules such as PRF, GRZs or FasL has been reviewed earlier by Luzio and colleagues (71). Briefly, these three effectors which presumably target to the same lytic granule compartment undergo different posttranslational modifications for their association with secretory lysosomes (80). GRZs are modified with a mannose-6-phosphate like many other proteins targeted to lysosomes (e.g. lysosomal hydrolases). In contrast, PRF is not modified with mannose-6-phosphate and rather receives complex glycans that presumably target the protein to granules by a yet unknown mechanism (80). In addition, also the lysosomal marker protein Lamp-1 has been suggested to play a role in the delivery of perforin to lytic granules (81). The transmembrane death factor FasL is also sorted to secretory lysosomes (**Figure 3**), although the subcellular entity may differ from the storage compartment for GRZ and PRF along with the ultimate subcellular destination of FasL as a transmembrane death ligand (6). In case of FasL, the crucial sorting motif is a unique polyproline stretch (25) that mediates binding to numerous SH3 domain proteins (82–89). It was shown in different studies that some of these interactors govern lysosome targeting or retention (84, 86, 90), whereas others mediate lipid-raft association (85) or targeting to the immunological synapse (87) thereby regulating storage, transport, surface appearance and function of the death factor in a rather complex fashion (91, 92). In addition, FasL interacts with Src kinases and tyrosine phosphorylation of FasL modulates its targeting to intracellular storage compartments (93). The proline-rich stretch of FasL is flanked by a dileucine motif that can be mono-ubiquitinated to facilitate subsequent sorting to intraluminal vesicles (ILV) (25, 93). In the case of granulysin, signals required for sorting are still unknown. As mentioned, 9 kDa GNLY emerges from the proteolytic cleavage of 15 kDa GNLY although the responsible protease has not been identified yet. However, this conversion can be blocked by Concanamycin A, an inhibitor of the vacuolar H^+^-ATPase that raises the pH in cytolytic granules (94). Thus, the differential localization of the 15 kDa and 9 kDa form of GNLY might rely on the presence or activity of the responsible protease and is linked to the pH of the storage compartments.

It is important to mention that the killing machinery of CTL and NK cells is highly efficient when cytotoxic cells face their target cells. Because their killing is so effective and to avoid collateral damage, it is crucial to restrict the release of cytolytic molecules to the immunological synapse forming between CTL or NK cell and their target. Thus, the centrosome of the cytotoxic cells, which serves as the main microtubule-organizing center (MTOC), locates to the plasma membrane at the contact site. Upon target recognition and binding, the secretory lysosomes are moved along microtubules in the (–)-direction toward the relocated MTOC at the synapse by the motor protein dynein, then dock and deliver their contents toward the target cell (6, 71, 95). However, especially the late steps of granule exocytosis including the final transport from the MTOC to the plasma membrane are still not very well characterized. A complex of RAB27A and Slp3 together with the (+)-end transporting microtubule motor protein kinesin-1 was recently suggested to drive lytic granules to the plasma membrane for ultimate secretion (96). In human NK cells, also transport along actin fibers by the motor protein Myosin IIa has been implicated in the release of secretory granules at actin hypodensities within the IS (97, 98). In addition, although RAB27A and MUNC13-4 play independent roles in activation-induced maturation of lytic granules (99), the Rab27A-MUNC13-4 complex is subsequently required to tether lytic granules to the plasma membrane for secretion (100).

It should be mentioned that the precise knowledge of lysosomal biogenesis, transport and release was also achieved by investigating the molecular causes and consequences of genetic defects associated with rare lysosomal storage disorders such as familial hemophagocytic lymphohistiocytosis (FHL) or Griscelli-, Hermansky-Pudlack and Chédiak-Higashi syndromes [see (71, 101) for review]. Since in several of these diseases (i.e. in the latter three syndromes), immunodeficiency coincides with albinism, this provided important information linking the biogenesis of secretory lysosomes or LREV from cytotoxic cells to lysosome-related organelles in melanocytes (71). As mentioned before, at certain phases of CTL/target cell interaction, the release of individual effectors seems to depend on distinct signal qualities that define non-classical (Ca^2+^-independent) or classical (Ca^2+^-dependent) degranulation, which may be governed by signal thresholds or antigen density on the target cell (7, 22, 36). How this selective mobilization is achieved mechanistically in terms of induced secretion of individual LREV or LRO, is, however, not precisely understood to date.

Hessvik and Llorente (74) and Gurunathan and colleagues (78) have reviewed the current knowledge on exosome biogenesis and release, and on their functions and therapeutic or clinical implications. For an overview on more historic aspects regarding the conceptual change in exosome research and the development of an almost exponential interest in exosomes in the last decade, we refer to the very recent article of Bassan and colleagues (76). More specific information on exosomes derived from lymphocytes is available in the recent reviews by Anel and colleagues (67) and by Calvo and Izquierdo (75). Regarding the biogenesis of exosomes, it reads like a blueprint of the biogenesis of LREV or LRO described above. According to Hessvik and Llorente, to release exosomes, several cellular steps need to be completed that indeed are likewise described for the generation of LREV/LRO: i.) the formation of intraluminal vesicles (ILVs) in MVBs, ii.) the directed transport of MVBs to the plasma membrane, and iii.) the fusion of MVBs with the plasma membrane for the release of ILVs as exosomes (74). As stated before for LROs, exosome biogenesis starts with early endosomes that mature into late endosomes/MVBs. Associated with endosomal membrane invagination, ILVs form and accumulate in the lumen of the MVB (102). These processes (as in LREV) are controlled by proteins of the endosomal sorting complex required for transport (ESCRT) family (103) and many other proteins including tetraspanins, syntenin, syndecan, diverse Rab proteins and many more [reviewed in (74)]. In fact, an earlier study by Stuffers and colleagues suggested that MVB can also form in the absence of ESCRT proteins (104) and that the selectively enriched tetraspanins might play a role in ESCRT-independent exosome release for instance in B lymphocytes (105). Notably, the recent proteomic screen by Kugeratsky and colleagues (69) supported these observations in a way and suggested that molecules governing biogenesis (including syntenin-1, ALIX and TSG101) might serve as the most reliable universal biomarkers for exosomes. However, they also pointed to the fact that often-used exosome markers like the tetraspanins CD9 or CD63 might not be reliable for exosome identification from all cell types, especially since lymphocyte exosomes apparently lack CD9 (69). Taken together, the biogenesis of exosomes (and LRO/LREV) has been described as partially ESCRT-dependent or -independent generation of ILV-loaded MVB. However, it is still open whether individual steps of vesicle formation or loading work synergistically or separately to also end-up in the formation of exosome subpopulations as we would suggest it for the different LREV (15, 106, 107). In addition, formation and release of exosomes or LREV is cell-type-specifically activation-dependent. Especially in CTL and NK cells, this safeguard mechanism and the focal release of LREV is prerequisite to avoid collateral damage.

Regarding the sorting of cargo molecules, exosomes seem to be more versatile since they contain different proteins, lipids and nucleic acids species. However, this assumption might be due to the different goal settings of the performed studies. If one for instance compares proteome analyses of intra- and extracellular vesicles for individual cell populations, common features become more apparent (69). In this regard, features of T-cell-derived exosomes display a high degree of overlap to LREV (67). In fact, one consequence of degranulation is the secretion of small ILV at the CTL-target cell synapse (4, 108). Thus, LREV degranulation might be regarded as a local release of small extracellular vesicles and although the ILV secreted by CTL were not referred to as exosomes at that time, their formation and mode of exocytosis would justify this classification. Early reports, however, described that re-stimulation of T cell blasts to induce activation-induced cell death (AICD) led to a non-directional secretion of EVs carrying pro-apoptotic FasL and Apo2L *via* MVB-mediated degranulation (109, 110), providing an alternative mechanism of TCR-controlled AICD without close cell-to-cell contact. Shortly after, these cytotoxic FasL-vesicles were indeed called “lethal exosomes” (111). It was also shown that upon TCR triggering, T lymphoblasts secrete exosomes containing intact TCR/CD3zeta complexes (66).

Interestingly, the MTOC positioning required for LREV movement to the immunological synapse is initially guided by a diacylglycerol (DAG) gradient centered at the contact area (112). DAG is generated by TCR-stimulated phospholipase C (PLC) and DAG phosphorylation by diacylglycerol kinase is involved in the spatiotemporal control of the activation-dependent DAG gradient (113) and thus MTOC polarization to the cytotoxic IS (112). In addition, DAG activates, among others, several members of the PKC and PKD families (114), such as PKCδ, which in turn is necessary for the polarization of lytic granules and cytotoxicity at least in mouse CTL (115, 116). One might now speculate that PKC activation might be sufficient to recruit the first set of LREV that contain FasL or 15 kDa GNLY (7, 22), whereas the release of the fully equipped LREV or dense granules containing GRZ, PRF and 9 kDa GNLY requires additional calcium signals.

In view of the potential overlap of LREV and EV (especially exosomes), we have recently started to directly compare the two described populations of LREV isolated by iodixanol gradients with exosomes purified by ultracentrifugation (UC) originating from split CD4^+^, CD8^+^ and γδ T-cell blast populations (unpublished data). In this thesis, exosomes and LREVs derived T cells were analyzed. UC-enriched exosomes were characterized by Western blotting, electron microscopy and nanoparticle tracking analysis (NTA) and flow cytometry according to the ISEV guidelines (63). LREV were enriched in parallel from the same cell populations as described (23). Differences and similarities between the protein content of both types of LREV and exosomes were investigated by Western blotting and two-dimensional difference gel electrophoresis (2D-DIGE). Western blotting revealed the presence of the exosome marker CD63 in all three types of vesicles. However, the other markers CD9 and CD81 could not be detected, indicating that these might not be suited for T-cell exosome characterization (69). With regard to GNLY, we confirmed the association of the 15 kDa form with light LREV, while the 9 kDa form was enriched in dense LREV. Interestingly, we detected the 15 kDa GNLY in exosomes during our study. Similarly, CD26/DPP4 was found in all three types of vesicles. Subsequent analyses of exosomes by flow cytometry confirmed these results. Moreover, in all T-cell subsets, exosomes were consistently more similar to the fraction of light LREV. Additionally, more similarities were found between the two types of LREV than between dense LREV and exosomes, suggesting that the light LREV and exosomes might share more common pathways during biogenesis and loading. Interestingly, constitutively released exosomes and exosomes released upon TCR ligation showed a very high degree of similarity (up to 95%) in the 2D-DIGE experiments, with only a few protein spots being increased in the exosomes after stimulation. Assuming that intact LRO/LREV are supposed to exhibit an additional membrane and luminal space this further substantiates the notion that exosomes indeed represent the ILV of LRO/LREV although transmembrane proteins might be underrepresented in 2D-DIGE analyses.

## Extracellular Vesicles and Tumor Immune Surveillance

It has early been noted that extracellular vesicles modulate the interplay between tumors, the tumor microenvironment and the immune system in different directions, occasionally resulting in opposing effects ranging from immune escape to effective tumor surveillance. This diverse impact of individual EV populations is presently addressed in many different studies on a wide range of tumor entities. Common aim of all these efforts is to better understand the relationship of EVs and associated cell populations in order to be able to manipulate the local environment for better effector function and immunosurveillance. Several reports showed that dendritic cell-derived exosomes may present cancer peptides to B, T and NK cells to elicit an immune-response, whereas immune cell-derived exosomes might also promote tumor progression in certain scenarios. On the other hand, tumor-cell derived EVs (TDEs) also display immunomodulatory properties that target both the effector and antigen-presenting arms of the immune system. However, although some studies show that TDEs can stimulate the immune system, they are predominantly considered immunosuppressive [reviewed in (117)]. As an example, metastatic melanoma release PD-L1^+^ EVs that suppress CD8^+^ T-cell function and thus promote tumor growth (118). In addition, tumor cells can downmodulate cell surface expression of the NKG2D ligands MHC class I–related chain (MIC) A and MICB and thus escape recognition by T and NK cells (119–121). In this scenario, the most frequent MICA allele *008 is released from tumor cells in association with EVs (120, 121) and treatment of NK cells with MICA*008^+^ EVs induced the downregulation of NKG2D from the cell surface and decreased NK cell cytotoxicity independent of NKG2D ligand expression on target cells (121). Microvesicles isolated from sera of newly diagnosed ALL patients also carried MICA/B, down-regulated expression of NKG2D in normal natural killer cells and decreased natural killer cell cytotoxicity. These microvesicles also displayed elevated levels of transforming growth factor-β (TGF-β) and neutralizing anti-TGF-β antibodies inhibited microvesicle-mediated suppression of NK cell activity and NKG2D down-regulation (122).

In terms of cytotoxic effector functions, especially NK cell-derived extracellular vesicles display direct tumoricidal properties and complement the arsenal of cytotoxic effector mechanisms. EVs harvested from cultures of ex vivo expanded NK cells contain the cytotoxic effector proteins PRF, GRZA, GRZB, GNLY and FasL and kill ALL cells and a neuroblastoma cell line *in vitro* by triggering different cell death pathways (123). Zhu et al. showed that NK-92 cell-derived exosomes carrying the effector molecules FasL, PRF and TNF-α decrease the viability and proliferation of melanoma cells (124). Besides cytotoxic effectors, Di Pace and colleagues showed that the cytotoxic activity of NK-cell derived exosomes is also linked to the presence of the adhesion protein DNAM1 on the exosome surface indicating that DNAM1 might increase the binding to and/or the internalization into tumor cells to facilitate apoptosis (125). Although the intracellular secretory compartments that give rise to exosomes are supposed to be more or less the same in T and NK cells, T-cell derived EVs have so far been less well characterized with respect to cytotoxic effector potential, but *in vitro* studies have shown that TCR stimulation facilitates EV release (66). Interestingly, T-cell activation seems to induce the differential release of distinct populations of EVs (126) which might at least in part be explained by the existence of different intracellular MVBs.

The expression of chimeric antigen receptors (CAR) provides effector T cells with tumor-targeting capabilities and CAR-based T-cell adoptive immunotherapy is a promising therapy for cancer. However, in about two thirds of patients the uncontrolled release of cytokines from CAR T cells leads to the cytokine release syndrome (CRS) that is characterized by nausea, headache, tachycardia, hypotension and rash. In severe cases, a cytokine storm might result in organ failure and death. Unfortunately, CAR T cell-associated toxicity cannot be controlled by simply reducing drug dosage [reviewed in (127)].

Recent evidence suggests that exosomes derived from CAR T cells might, however, facilitate the anti-tumor response which could allow for a switch to cell-free protocols to minimize adverse effects and at the same time could easily be adjusted with respect to the applied dose. It was shown that CAR T cell derived exosomes display the CAR T-cell receptor on their surface, contain GRZB and PRF and selectively induce cell death in targeted tumor cells *in vitro* and reduce tumor growth *in vivo* (128). Thus, exosomes derived from CAR T cells targeting mesothelin showed surface expression of the respective CARs and CD3 and inhibited the growth of mesothelin-positive triple-negative breast cancer cells which, might be attributed to tumor cell killing by PRF and GRZB. Anti-tumor effects of these CAR T-cell exosomes were also observed *in vivo* without apparent adverse effects (129). In another approach, an anti-CD19 scFv was fused with CD63 to generate CD19-targeting exosomes as a drug delivery system. As a proof of principle, these exosomes were loaded with doxorubicin and showed improved cytotoxicity to mantle cell lymphoma cells when compared to doxorubicin alone (130).

Although the contribution of effector cell-derived EVs to immunosurveillance and clearance of tumors is not characterized in detail, these few examples already highlight the potential of extracellular vesicles to facilitate effector function in tumor immunotherapies employing adoptive cell transfer or to even switch to cell-free protocols.

## Conclusion and Challenge

Although over the last two decades, studies on intracellular LRO or LREV and on exosomes have often been conducted as different branches, the overall similarities and common pathways of the biogenesis, transport and release of such vesicles might indicate that we are dealing with highly related organelles and that ILV of LRO/LREV actually resemble exosomes. The most challenging questions often asked when mentioning exosomes are: “What are they good for?” or more specifically “Why should a cell release such vesicles with supportive or dangerous material in an untargeted manner, if local transfer of effector molecules is much more specific/safe and presumably also more effective?”. This holds especially true in the case of cytotoxic effector vesicles released from CTL and NK cells in a contact-restricted manner into the IS.

## Author Contributions

Conceptualization, ML, OJ. Writing—original draft preparation, OJ. Writing—review and editing, ML, OJ. Visualization, ML, OJ. Funding acquisition, OJ. All authors have read and agreed to the published version of the manuscript.

## Funding

This work was supported by the Deutsche Forschungsgemeinschaft (DFG, grant JA 610 7/3), the Medical Faculty of the University of Kiel and the Cluster of Excellence ‘‘Inflammation-at-Interfaces’’.

## Conflict of Interest

The authors declare that the research was conducted in the absence of any commercial or financial relationships that could be construed as a potential conflict of interest.

## Publisher’s Note

All claims expressed in this article are solely those of the authors and do not necessarily represent those of their affiliated organizations, or those of the publisher, the editors and the reviewers. Any product that may be evaluated in this article, or claim that may be made by its manufacturer, is not guaranteed or endorsed by the publisher.
